# Occupational exoskeletons: A roadmap toward large-scale adoption. Methodology and challenges of bringing exoskeletons to workplaces

**DOI:** 10.1017/wtc.2021.11

**Published:** 2021-09-17

**Authors:** Simona Crea, Philipp Beckerle, Michiel De Looze, Kevin De Pauw, Lorenzo Grazi, Tjaša Kermavnar, Jawad Masood, Leonard W. O’Sullivan, Ilaria Pacifico, Carlos Rodriguez-Guerrero, Nicola Vitiello, Danijela Ristić-Durrant, Jan Veneman

**Affiliations:** 1 Scuola Superiore Sant’Anna, The BioRobotics Institute, Pontedera, Italy; 2 IRCCS Fondazione Don Gnocchi, Florence, Italy; 3 Chair of Autonomous Systems and Mechatronics, Friedrich-Alexander-Universität Erlangen-Nürnberg, Erlangen, Germany; 4 Institute for Mechatronic Systems, Technische Universität Darmstadt, Darmstadt, Germany; 5 TNO, Leiden, The Netherlands; 6 Human Physiology and Sports Physiotherapy Research Group, and Brussels Human Robotics Research Center (BruBotics), Vrije Universiteit Brussel, Brussels, Belgium; 7 School of Design, and Confirm Smart Manufacturing Centre, University of Limerick, Limerick, Ireland; 8 Processes and Factory of the Future Department, CTAG – Centro Tecnológico de Automoción de Galicia, Pontevedra, Spain; 9 Robotics and Multibody Mechanics Research Group, Department of Mechanical Engineering, Vrije Universiteit Brussel and Flanders Make, Brussel, Belgium; 10 Institute of Automation, University of Bremen, Bremen, Germany; 11 Chair of COST Action 16116, Hocoma Medical GmbH, Zürich, Switzerland

**Keywords:** field studies, industrial ergonomics, occupational exoskeletons

## Abstract

The large-scale adoption of occupational exoskeletons (OEs) will only happen if clear evidence of effectiveness of the devices is available. Performing product-specific field validation studies would allow the stakeholders and decision-makers (e.g., employers, ergonomists, health, and safety departments) to assess OEs’ effectiveness in their specific work contexts and with experienced workers, who could further provide useful insights on practical issues related to exoskeleton daily use. This paper reviews present-day scientific methods for assessing the effectiveness of OEs in laboratory and field studies, and presents the vision of the authors on a roadmap that could lead to large-scale adoption of this technology. The analysis of the state-of-the-art shows methodological differences between laboratory and field studies. While the former are more extensively reported in scientific papers, they exhibit limited generalizability of the findings to real-world scenarios. On the contrary, field studies are limited in sample sizes and frequently focused only on subjective metrics. We propose a roadmap to promote large-scale knowledge-based adoption of OEs. It details that the analysis of the costs and benefits of this technology should be communicated to all stakeholders to facilitate informed decision making, so that each stakeholder can develop their specific role regarding this innovation. Large-scale field studies can help identify and monitor the possible side-effects related to exoskeleton use in real work situations, as well as provide a comprehensive scientific knowledge base to support the revision of ergonomics risk-assessment methods, safety standards and regulations, and the definition of guidelines and practices for the selection and use of OEs.

## Introduction

Occupational exoskeletons (OEs) can be defined as personal assistive devices that can reduce the physical load on workers performing demanding activities, by acting synergistically with their users (Monica et al., 2020). Currently, most OEs are designed either to support specific body parts or to augment specific human capabilities. Regarding the former, the majority of OE devices have been developed to reduce the physical load on the upper limbs and the lumbar region of the spine, whereas support of other body parts, such as the knees or wrists, have been less explored.

As work-related musculoskeletal disorders (MSDs)^1^ are associated with high costs to the employers, either related to direct compensation costs or indirect costs (e.g., lost wages, lost production, cost of recruiting and training replacement workers, and healthcare costs for rehabilitating the affected workers), companies have shown interest in OEs as possible solutions to reduce exposure of their workers to physical risk factors that can cause MSDs, and alternatives and/or complementary aids to more expensive solutions for reducing physical strain on their workers (such as collaborative robots). Hence, the main long-term expectations of companies on OEs are the reduced occurrence of work-related MSDs and related improved productivity (due to reduced absence rate) (Howard et al., 2020; Monica et al., 2020).

Motivated by the large potential to improve the ergonomics of workstations in their production lines, car manufacturers, in particular, have pioneered research in using the robots at workplaces. In this field, despite the extensive use of robots in most welding, painting, and assembly operations, final assembly lines still require several manual operations, such as the assembly tasks of the car interior, underbody, doors, windows or trims, and sealing of the doors. These are performed at a high pace (work cycles of a few minutes for small cars), and in some cases require maintaining awkward postures while performing high-precision actions. Recently, several car manufacturers have evaluated and tested exoskeletons within their plants, in some cases developing custom devices to meet their specific needs (Spada et al., 2017; Gillette and Stephenson, 2018; Claramunt-Molet et al., 2019; Ferreira et al., 2020; Iranzo et al., 2020). In addition to the automotive field, exoskeletons have been proposed in various other manufacturing contexts, as well as in nonmanufacturing domains, such as logistics, construction, agriculture, and patient care. However, currently, the actual adoption of exoskeletons in real scenarios is limited compared to the expectations.

Such a limited-scale adoption of OEs may be related to different factors. One of them is the lack of clear evidence of adequate effectiveness of the devices in the final workplaces. While system capability to reduce the load on specific body parts in stereotyped gestures could be investigated by OE manufacturers in laboratory settings (*in-lab*), the device effectiveness is use-case specific and therefore should be studied in dedicated experiments: use-case specific experiments could be carried out in laboratory-reconstructed workstations or directly at the individual workstation (*in-field*). Performing product-specific validation studies *in-field* would allow the stakeholders and decision-makers to assess the effectiveness of devices in their specific work contexts and with experienced workers who could provide useful insights into practical issues related to exoskeleton use in daily practice. In such field studies, effectiveness indices may include experience-related indicators, measured through questionnaires and structured interviews, as well as instrumental parameters, based on kinematics, kinetics, and physiological (e.g., cardiovascular, pulmonary, and muscular) measures (Ármannsdóttir et al., 2020; Torricelli et al., 2020).

This paper aims to provide a comprehensive overview of the scientific methods used to investigate the effectiveness of OEs, comparing the methods used in *lab* and *field* conditions. Also, the paper presents the position of the authors suggesting a roadmap that might support large-scale adoption of the technology. The following sections are organized as follows. Section “Technological Approaches to Design OEs” introduces different OE technologies, with a particular focus on their classification based on different kinematics and actuation approaches, and the regulatory-based classification, since these aspects are relevant, together with other aspects, for assessing the effectiveness of the devices in different contexts. Section “Scientific Evidence of OE Effectiveness in Laboratory and Field Studies” provides a narrative overview of the state-of-the-art of methodological approaches to validate exoskeletons in laboratory and field studies. Finally, section “Roadmap Toward Large-Scale Adoption of OEs” outlines a proposed roadmap for large-scale adoption of OEs, including the process of implementing OEs into specific workplaces, communication with different stakeholders, and future perspectives.

## Technological Approaches to Design OEs

**Figure 1.**
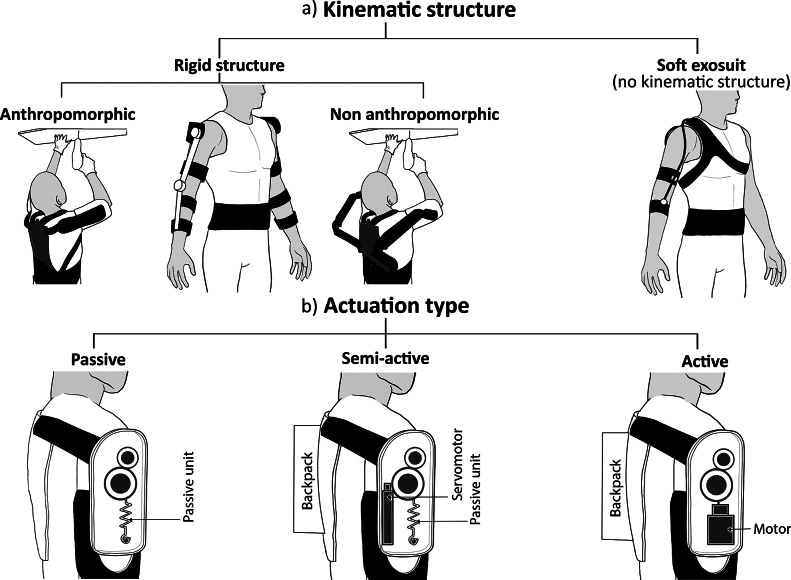
Occupational exoskeletons classification based on kinematic structure (a) and type of actuation (b). Concerning the kinematics, rigid-structure devices can be classified in anthropomorphic and nonanthropomorphic devices, whereas soft exosuits do not present any kinematic structure. Actuation types include passive, semi-active, and active systems. In the picture actuation types are depicted for an anthropomorphic device. Sketches are provided for upper-limb devices.

Industrial scenarios and workflows tend to be very dynamic and diverse, and workers rarely continuously repeat a limited set of movements. Taking this into account, OEs should be designed to comply with very diverse conditions of use while finding the most appropriate trade-offs to fulfill various requirements (e.g., lightweight design and system *adaptability*). This is a challenge for exoskeletons designers. Several design architectures have been proposed to realize compact, lightweight, and effective OEs by following diverse approaches, mostly in terms of kinematic structure and type of actuation (de Looze et al., 2016; Fox et al., 2019; Toxiri et al., 2019; Gull et al., 2020; Huysamen et al., 2020; Kermavnar et al., 2020). The classification of OEs in terms of kinematics structure and actuation is illustrated in Figure 1.

The *kinematic* compatibility of a wearable device is paramount for a comfortable fit of a wearable device, effective torque transfer, and minimization of undesired forces to the human skeletal system. The exoskeleton should allow the user to move within his/her physiological range of movement and must account for the translations associated with the joint rotational movements (Voilqué et al., 2019; Rossini et al., 2021). Rigid kinematic structures of upper-limb OEs typically originate from a frame attached to the pelvic region of the human body and end at the arm attachment point. Lower-back exoskeletons, instead, usually have rigid kinematic structures surrounding the user’s hips and lower back, with attachment points located in correspondence with the thighs and the trunk. Based on their *kinematic structures*, rigid exoskeletons can be classified as anthropomorphic or nonanthropomorphic (e.g., end-effector-like) devices (Schiele and van der Helm, 2006; Naf et al., 2018). Exoskeletons with anthropomorphic kinematic structures include robotic joints that need to be aligned with the user’s joint axes, thus misalignment-compensation strategies should be included to counteract the effects of axis misalignments; whereas exoskeletons with nonanthropomorphic kinematic structures do not require a direct correspondence between the robot’s and user’s axes of rotation (Nef et al., 2009; Accoto et al., 2014). Regarding upper-limb exoskeletons, anthropomorphic kinematic structures typically have the shoulder-elevation axis collocated in correspondence with the shoulder glenohumeral joint, and related misalignment compensation strategies based on passive degrees of freedom and/or compliant elements. Back-support exoskeletons typically exert assistive force around the human hips and the lumbosacral joint (i.e., L5/S1 joint), respectively with anthropomorphic and nonanthropomorphic approaches due to the difficulty in identifying and accessing the L5/S1 joint specifically. Different from rigid-structure devices, soft exosuits are wearable clothing-like devices that can generate moments around biological joints through pulling cables and textiles acting in parallel to the action of muscles and tendons. In these systems compressing loads are not sustained by any external rigid structure but are sustained by the wearer’s bone structure. Lumbar exosuits apply assistive forces in parallel with the lumbar and hip extensor muscles, with elastic textiles and/or cables running along the back and thighs (Lamers and Zelik, 2021), whereas upper-limb exosuits assist the shoulder and/or elbow flexors (Kim et al., 2020a).

As far as *actuation* is concerned, OEs can be classified into three subcategories: active, passive, or semi-active exoskeletons. Active exoskeletons use powered actuators to generate assistive torque and rely on sensors and control units to synchronize robot action with the user’s motion, whereas passive exoskeletons exploit springs or spring-like elements to store and release energy in various phases of the human movement (de Looze et al., 2016). The comparison of these two actuation alternatives should account for multiple factors. Currently, passive OEs have higher maturity compared to their active counterparts, as their functioning does not involve the use of actuators, batteries, wiring, and electronics and their physical human–robot interface has a repeatable and intuitive behavior. In very dynamic and diverse operating environments, active OEs can be more adaptable than passive devices in different tasks, but the need for extremely accurate control algorithms currently prevents their use in field scenarios (De Bock et al., 2020). To increase system adaptability and with the final objective to further increase the system acceptance, mechanical clutches and null-assistance configurations have been implemented into some passive devices: they usually require some type of human intervention to engage or disengage the mechanisms to allow for larger freedom of movement while not executing the specific tasks they were designed for (e.g., walking or grasping tools) (Baltrusch et al., 2020c). This may end up being cumbersome and impractical in the field, especially when manual tasks are involved. In the attempt to achieve the trade-off between the larger adaptability of active systems and greater usability of passive devices, a trade-off could be represented by semi-active exoskeletons that use low-power servo motors to adapt the behavior of the device based on the user’s needs, for example, by adapting the level of assistance or engaging/disengaging the actuation mechanisms (Figure 1).

In addition to considerations of the actuation architecture, the comparison of the actuation of different OEs should consider the amount of assistive torque the devices can exert on the target body parts. The ratio between the assistive torque and the physiological joint torque exerted by muscles in a specific task could be computed to provide an estimate of the biomechanical unloading due to the use of the device. The physiological torque exerted by lower-back extensor muscles around the L5-S1 joint can reach up to 200 Nm during trunk flexion and extension movements without handling tools, with an angular velocity in the range of about 90–120 deg/s (and peak power of 0.7–1.1 W/kg) (Saraceni et al., 2021), or up to 300 Nm when lifting a 15-kg load (Koopman et al., 2019a), whereas shoulder elevator muscles typically exert 30 Nm when keeping the arms overhead holding tools such as a drill (Anton et al., 2001). To provide sufficient assistive torque to reduce the physiological workload on the lumbar or shoulder joints, most devices are designed to output torques in the range of 10–20 Nm per hip or up to 40 Nm at the back (Huysamen et al., 2018b; von Glinski et al., 2019; Lanotte et al., 2021) and 3–6 Nm for the shoulder (de Vries et al., 2019; Grazi et al., 2020), spanning between the 5 and 20% of the physiological joint torque.

Example of state-of-the-art OEs classified according to their kinematics or actuation is given in Table 1.

**Table 1.** Classification of occupational exoskeletons (OEs) according to kinematics, structure, and actuation; within each category, a nonexhaustive list of commercial and prototypical OEs is given

Kinematics					
Upper-limb exoskeletons			Back-support exoskeletons		
Rigid structure		Soft exosuits	Rigid structure		Soft exosuits
Antropomorphic kinematic chain	Nonantropomorphic kinematic chain	No kinematic chain	Antropomorphic kinematic chain	Nonantropomorphic kinematic chain	No kinematic chain
MATE^TM^ ShoulderX^TM^ Levitate^TM^ EksoVest^TM^	Paexo Shoulder^TM^ Besk G^TM^	Zhou et al., 2021	ATOUN MODEL Y^TM^ Paexo Back^TM^ HAL^TM^ for Labor Support (von Glinski et al., 2019) Cray X^TM^		Lamers and Zelik, 2021 Lamers et al., 2018 PLAD (Abdoli-E et al., 2006)

Actuation					
Upper-limb exoskeletons			Back-support exoskeletons		
Passive	Semi-active	Active	Passive	Semi-active	Active
MATE^TM^ ShoulderX^TM^ Levitate^TM^ Paexo Shoulder^TM^ EksoVest^TM^ Skelex^TM^	H-PULSE (Grazi et al., 2020)	MovilFrio (Blanco et al., 2019) ExIF (Blanco et al., 2020) Lucy 2.0 (Otten et al., 2018)	Back-X^TM^ (Kazerooni et al., 2019) Spexor (Näf et al., 2018) Laevo^TM^ PLAD (Abdoli-E et al., 2006)	SPEXOR (Jamšek et al., 2020) Exosuit (Lamers and Zelik, 2021)	APO (Lanotte et al., 2021) Robo-Mate (Huysamen et al., 2018b) H-WEX (Ko et al., 2018) AB-Wear (Inose et al., 2017) iWear (Lanotte et al., 2020) HAL^TM^ (von Glinski et al., 2019) XoTrunk (Poliero et al., 2020)

## Scientific Evidence of OE Effectiveness in Laboratory and Field Studies

A search of electronic and personal databases was performed to identify papers describing objective and subjective evaluations of OEs in *laboratory* and *field* studies. Methodological aspects of exoskeleton testing were reviewed for active and passive back-support and upper-limb devices. In total, 99 studies were analyzed and are listed in the Supplementary Materials. A summary of studied tasks and effects of exoskeleton use is provided below.

### Laboratory Studies

The main goal of laboratory studies is to validate OEs, and to investigate their biomechanical effects on the users’ physical effort, or the level of assistance they can provide. An additional goal of laboratory studies is to identify potentially undesired effects (e.g., due to load redistribution on the human body) during the tasks under investigation. Biomechanical studies carried out in laboratory conditions can benefit from the use of motion tracking systems, force platforms, or high-density EMG recorders, which would be impractical to use outside the laboratory.

The potential of back-support and upper-limb exoskeletons to reduce the mechanical loads in the back and shoulder regions has been frequently addressed in laboratory studies (McFarland and Fischer, 2019; Theurel and Desbrosses, 2019; de Vries and de Looze, 2020; Howard et al., 2020; Kermavnar et al., 2020).

#### Back-support exoskeletons

A total of 57 laboratory studies of back-support exoskeletons were identified (Supplementary Table S5).

The devices were typically tested during static bending or lifting tasks (including single lifts and repetitive lifting), and less often during complex tasks that more closely resembled real-work situations.

Studies of static bending with OEs tended to involve passive devices, tested on healthy young or middle-aged participants who were predominantly male. The bending position was typically determined by trunk flexion angle (40–55°; Bosch et al., 2016; Wei et al., 2020a), or hand distance from the floor (Kim et al., 2020b). The bending duration was either decided in advance, ranging from 5 to 300 s (Koopman et al., 2020a; Wei et al., 2020a, 2020b), or determined by the participants’ perception of discomfort (Bosch et al., 2016). The studied effects most commonly included EMG activity of back, abdominal, upper- and lower-limb muscles, metabolic cost, various joint angles, and ground-reaction forces (Bosch et al., 2016; Koopman et al., 2019a, 2020a; Kim et al., 2020b; Wei et al., 2020a, 2020b). Among subjective measures, local discomfort, perception of balance, device fit, comfort, body movement constraints, and overall helpfulness of the OE were assessed (Bosch et al., 2016; Kim et al., 2020b).

Single symmetric and asymmetric lifts were performed with active and passive back-support exoskeletons, and also predominantly involved healthy young and middle-aged male participants. OE target users in the luggage-handling sector of the airline industry were included in one study (Koopman et al., 2020a). The starting position of the loads ranged from the floor to 10 cm above the knee, and they were typically lifted to waist height, in many cases including trunk rotation (Chen et al., 2018; Huysamen et al., 2018b; Lanotte et al., 2018; Toxiri et al., 2018; Alemi et al., 2019; Goršič et al., 2019; Koopman et al., 2019b, 2020a, 2020b; Picchiotti et al., 2019; Hussain et al., 2020; Hyun et al., 2020). Loads ranged from 5 through 18 kg across the studies and were lifted using different techniques at a comfortable pace, or every 6–15 s. The EMG activity of back, abdominal and lower-limb muscles were most investigated, together with various joint angles, ground-reaction forces, lower-back extension moments and/or compression, trunk extension time, and contact pressure. Among subjective measures, perceived exertion, local discomfort, musculoskeletal pressure, and exoskeleton usability were assessed in some studies (Huysamen et al., 2018b; Alemi et al., 2019).

Repetitive lifting was performed with active and passive exoskeletons, mainly by healthy young and middle-aged participants, predominantly men (Baltrusch et al., 2019; Heo et al., 2020; Madinei et al., 2020a, 2020b; Wei et al., 2020a, 2020b). One study included workers with low-back pain (Baltrusch et al., 2020a). Across the studies, loads of 6–20 kg were lifted during standing or kneeling, with the starting position ranging from the floor to knee level, and end position from 50 cm above the floor to hip height. Lifting was performed using different lifting techniques at 5–30 cycles/min, and lasted for 1–5 min or until exhaustion. Measured were the EMG activity of back, abdominal, upper- and lower-limb muscles, various joint angles, ground-reaction forces, number of lifting cycles until exhaustion, and metabolic cost (Baltrusch et al., 2019; Tan et al., 2019; Baltrusch et al., 2020b; Heo et al., 2020; Madinei et al., 2020a, 2020b; Wei et al., 2020a, 2020b). Subjective measures included perceived exertion, fatigue, balance, local discomfort, device fit, comfort, movement constraints, and overall usability (Tan et al., 2019; Madinei et al., 2020a, 2020b).

The literature demonstrates a very wide range of approaches to experimental design for assessing back-support exoskeletons. This variability among studies makes it difficult to directly compare the results. The first attempt toward a standardized testing approach was made with the development of a functional performance test battery which includes 12 tasks to assess the support and hindrance of the user by the exoskeleton (Baltrusch et al., 2018). The tasks supported by the exoskeleton include repetitive lifting (20-kg load from ankle height, 2 min), carrying (20-kg box, 10 m), static bending (a simple manual task at knee height, 30–60° trunk flexion, max. 5 min), and three-point kneeling (a simple manual task with one hand on the floor, max. 5 min). The tasks hindered by the exoskeleton include walking, sit-to-stand/stand-to-sit, climbing stairs and ladder. The tasks where the exoskeleton potentially affects the range of motion involve forward bending with extended knees, wide stance, trunk rotation, and squats.

Other testing scenarios have also been reported involving healthy, predominantly young participants. Passive back-support exoskeletons were tested during walking on a treadmill for 5 min or across the laboratory at preferred speed (Baltrusch et al., 2019; Goršič et al., 2019), and the EMG activity of back, abdominal, and lower-limb muscles was measured, as well as low-back extension moment and various joint angles (Baltrusch et al., 2019; Goršič et al., 2019). During standing up from a stool, the EMG activity of back and abdominal muscles, low-back extension moment, and trunk flexion angle were measured (Goršič et al., 2019). In sitting with perturbation from the front, back, left and right, trunk deflection and EMG activity of back and abdominal muscles were measured (Goršič et al., 2019). During repetitive pick-and-place tasks at 40° trunk flexion with occasional lifting of bins to shoulder height, the EMG activity of back, abdominal, and upper-limb muscles was measured, and local discomfort was assessed (Bosch et al., 2016). In some cases, the laboratory settings were a mock-up of the workplace environment (Baltrusch et al., 2020b), or simulated assembly work (Bosch et al., 2016).

Active exoskeletons were studied during snow shoveling as fast as possible until fatigued (Miura et al., 2018), and during a task composed of sit-to-stand, walking, lifting–lowering, walking back, and stand-to-sit (Chen et al., 2018). In the former case, the number of scoops, shoveling time and distance, heart rate, blood pressure, and lumbar fatigue perception were measured; and in the latter case, the EMG activity of back muscles was assessed.

#### Upper-limb exoskeletons

A total of 29 laboratory studies of upper-limb exoskeletons (Supplementary Table S3) were identified and analyzed.

Across the studies performed in a laboratory environment, the majority of exoskeletons subjected to research were passive while a few studies involved active exoskeletons.

Tasks performed in laboratories to investigate the effects of wearing an exoskeleton vary across studies. Most studies concentrate on tasks requiring arm elevation with varying elevation angles (roughly ranging from hands at waist level, in front of the body, up to overhead work) (de Vries et al., 2019). The main focus across the studies is on static tasks where the arms are elevated and held in a static position (Huysamen et al., 2018a, Schmalz et al., 2019; Grazi et al., 2020), or on quasi-static tasks, where a specific activity is performed with hardly any arm movement around the shoulder joint (Pacifico et al., 2020). These quasi-static tasks comprise (overhead) assembly (Perez Luque et al., 2020), drilling (Alabdulkarim and Nussbaum, 2019), wiring (Kim et al., 2018), and sanding (Moyon et al., 2018). The handled loads (e.g., hand tools) in these types of tasks range from 0 to 5 kg. More dynamic tasks that have been studied are carrying and stacking (Theurel et al., 2018) and plastering of ceilings and walls (de Vries et al., 2021).

Various variables have been used to assess the direct effect on shoulder load and the short-term human body response. For assessing the effect on workload, a variable that has been addressed in lab studies is the shoulder torque (e.g., de Vries et al., 2019), that is, the torque to be generated by the person when wearing an exoskeleton, estimated by inverse dynamics techniques. Levels of muscle activation have also been used in multiple studies as a parameter to evaluate the effect of an exoskeleton on shoulder load (e.g., Naito et al., 2007; Huysamen et al., 2018a; Kim et al., 2018; Van Engelhoven et al., 2018). Muscles activation has been assessed through EMG measurements. EMG measures were carried out on the arm elevator muscles, particularly the anterior deltoid, medial deltoid, trapezius pars descendens, and biceps brachii muscles, extensor muscles, like the posterior deltoid and triceps brachii, and shoulder stabilizers, such as the pectoralis major muscle. In addition to average activation, the assessment of muscle fatigue by frequency analysis has been considered in a few laboratory studies, despite the difficulty of controlling all possible confounding factors (Rashedi et al., 2014). Other variables have also been studied: endurance was studied by Spada et al. (2017), various kinematic variables (Maurice et al., 2019; Schmalz et al., 2019; Grazi et al., 2020; Pacifico et al., 2020) or physiological variables like heartrate or oxygen uptake (Theurel et al., 2018; Maurice et al., 2019; Grazi et al., 2020) have been investigated by researchers in different studies.

### Field Studies

The goal of exoskeleton evaluation in field studies is to determine their effects in the workplace settings on one or more of the following variables: (a) behavior, (b) use, usability, and acceptance, (c) performance, and (d) workload, discomfort, and fatigue. As the use of complex experimental equipment at the workplace would be impractical, instrumental measurement systems typically include wearable sensors, such as IMUs and wireless EMG recorders.

While studying the incidence of work-related MSDs (e.g., low back pain and neck-shoulder injury) could be considered a primary goal of field studies, it is important to understand that large long-term controlled studies have not been performed yet, likely due to the significant effort required and relatively low maturity of the market.

#### Back-support exoskeletons

In total, five field studies of back-support exoskeletons were identified (Supplementary Table S6).

Graham et al. (2009) performed a study of the Personal Lift Assist Device (PLAD) on healthy automotive operators during a highly standardized automotive assembly task. Two hours of 55-s cycles of manual work were monitored, with static bending and trunk rotation, standing, walking, and carrying of car parts. EMG activity of back and abdominal muscles, and joint angles were recorded.

The study by Amandels et al. (2019) of Laevo V2.4 was performed on middle-aged male shop-floor workers at a press and shear department with low-back pain. The participants were monitored 30 min per day for 3 weeks while performing their typical work tasks of frequent far-reaching with bending. Measures included EMG activity of the back, upper and lower-limb muscles, joint angles, perceived level of local discomfort, and users’ impression.

A 4-week field study of Laevo was performed by Hensel and Keil (2019) on 30 male employees in the automotive industry. The study was conducted at five workplaces in the assembly and press shop (ground screw connection footwell, trunk insulation, installation cable harness, maintenance, and press set up) with tasks performed in a marked static forward bend. In addition, three workplaces with considerable upper-body flexion in logistics were selected to evaluate dynamic-repositioning activities. Perceived relief at the lower back and discomfort, usability, and user acceptance were evaluated.

Motmans et al. (2019) performed a study of Laevo V2.5 on middle-aged male order-pickers of cheese with low-back pain. The participants’ EMG activity of back and upper-limb muscles, joint angles, and perceived device effect on work task, effectiveness, and comfort were measured during 1.5 hr of cheese picking.

Settembre et al. (2020) performed a field study of Laevo v1 on two male workers at a COVID-19 intensive care unit. The participants were equipped with the exoskeleton in compliance with the strict hygiene measures of the intensive care unit during the outbreak. During a typical 3-hr shift, each participant performed 10 prone positioning/supine positioning maneuvers that involved static forward bending to secure the patient’s head, forward trunk flexion during SP and forward trunk flexion during repositioning of the patient on the bed. The participants’ perceived physical effort and relief were measured, as well as their cardiac activity.

#### Upper-limb exoskeletons

In total, eight field studies of upper-limb exoskeletons were identified (Supplementary Table S4).

Gillette and Stephenson (2018) conducted a field study of Levitate Airframe during an overhead automotive assembly at Toyota Canada. Eleven experienced male workers performed 10 automotive assembly tasks. Measurements included EMG activity, fatigue, and injury hazard.

Moyon et al. (2018) conducted a field study of the upper-body passive exoskeleton Skelex at a boat-manufacturer worksite. Nine workers performed one of the finishing operations on a catamaran hull: sanding, coating, polishing, or painting. The measurements of heart rate, posture analysis, and usability were performed.

Gillette and Stephenson (2019) conducted a field study of Levitate Airframe on six workers performing tasks that involved prolonged elevated arm postures, including cab assembly, hydraulic assembly, parts painting, parts hanging, and frame welding tasks. The EMG activity of arm, shoulder, and lower-back muscles, and fatigue hazard were measured.

Smets (2019) conducted a field trial of EksoVest with 22 operators during overhead automotive assembly work. The study was performed in three phases: the first consisted of the collection of subjective first impressions and feedback on proto-EksoVest (eight operators); the second consisted of estimating the usability (10 operators); and the third included a 3-month use for the collection of usability and musculoskeletal discomfort evaluations (four operators).

De Bock et al. (2020) performed a field study in which four operators tested two commercial upper-limb exoskeletons (i.e., the ShoulderX and Skelex). The tasks consisted of transferring wind-screens from a trailer into a storage rack and then back to the trailer and transferring the wind-screens from the forklift platform to the storage rack and then back to the forklift platform. Muscular activity, heart rate, local discomfort, usability, and task load were assessed.

Hefferle et al. (2020) performed a field study of two different overhead exoskeletons, Crimson Dynamics and Skelex V1, with eight male associates on the assembly line of an automotive manufacturer. The workers performed their daily tasks at the workstation with a mean shoulder joint angle of 90° depending on employee stature. Perceived local strain of shoulders and neck, and whole-body strain were assessed.

Iranzo et al. (2020) performed a field study of Levitate Airframe on 12 workers (11 male, 1 female). The participants performed repetitive overhead work, assembling the car body from underneath the car with pneumatic screwdrivers. EMG activity was measured, as well as the angles of the neck, back, and upper-limb joints.

Wang et al. (2021) performed a field study of the passive upper-limb exoskeleton PULE with 8 farmers at the orchard farming test site. Four participants performed fruit thinning with the arms in a static elevated position (100° shoulder flexion), and four participants sprayed pesticide with the hands swinging from 0° to 100° and back. EMG activity and the angle of the upper limb were measured.

#### Main methodological differences between laboratory and field studies

**Figure 2.**
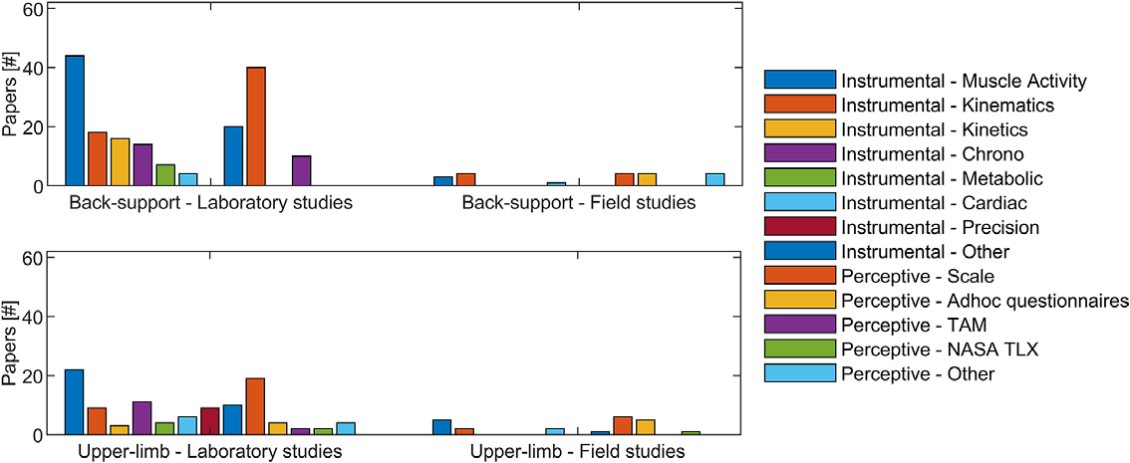
Frequency analysis of the objective and subjective metrics between laboratory and field studies for back-support and upper-limb exoskeletons. Acronyms: Technology Acceptance Model (TAM), NASA task load index (NASA TLX).

Figure 2 visualizes the metrics of back-support and upper-limb exoskeletons in laboratory and field conditions. Relatively few field studies have been conducted compared to laboratory studies, with back-support studies showing a ratio between field to laboratory studies equal to 1 to more than 10, and upper-limb studies showing a ratio of 1 to more than 3.

EMG and subjective measures (Rating of Perceived Exertion—RPE; System Usability Scale—SUS) are the most consistent metrics across field and laboratory studies. Indeed, the key measurement in laboratory studies is surface EMG signals are of interest for two reasons. Firstly, the forces that are generated by muscles around a specific joint directly lead (through *action–reaction*) to an increase in internal loading forces (compression and shear forces) on joint structures. Secondly, the level of muscle activation that is required for a specific task is a predictor of muscle fatigue development. Fatigue, in turn, could be assessed by a simultaneous temporal increase of the EMG amplitude and decrease of its frequency content (Basmajian and De Luca, 1985).

Another important parameter in the assessment of OEs is endurance, that is, the duration of a person’s capability to perform a specific task without exhaustion or before the onset of a predefined level of fatigue, evaluated by the perceived exertion on an RPE scale (Borg, 1982). Also, perceived effort, discomfort, and fatigue have been frequently used to evaluate OEs. Perceived discomfort and fatigue (in contrast with perceived effort) are variables that need time to develop and therefore require relatively long trials to get relevant results. The selection of metrics depends on whether the studies are conducted in laboratory or field conditions, with a tendency toward more instrumental data gathering in laboratory conditions.

Although studies in controlled laboratory environments involve multidisciplinary approaches to provide the bigger picture of exoskeleton functioning, field studies mainly include easy-to-monitor and easy-to-use measures. Field studies are of major importance to prove whether or not results from laboratory studies are generalizable to the field conditions. From a practical point of view, this is not straightforward, since body movements in occupational settings are task-specific and thus differ from simulated stereotyped tasks, such as static bending for a given amount of time, lifting a box using a specific lifting technique, or walking 10 m on level ground without obstacles. Additionally, in the field studies, the effectiveness of exoskeletons is also challenged beyond the sole functionality of the device, which compromises the transfer of research results from the laboratory to the field conditions. Current wearable sensors for biomechanics measurements may still be too complex to be used in field conditions. As it would increase the complexity to the setup of the study and the experimental procedures (e.g., due to the need for frequent calibration procedures), future advancements in wearable sensory systems (such as inertial measurement devices, portable pressure-sensitive insoles, mobile electro-physiological devices, and gas exchange devices) could enable gathering more instrumental data also in field studies.

In addition to gathering data, the assessment of the measurement systems and metrics extracted from lab and field studies revealed that only in a few studies, instrumental and perception-related assessment of OEs efficacy served as the basis for the evaluation on how the risk factors associated with a certain task changed. In particular, for back-support OEs, only 13 out of 62 lab and field studies used biomechanics measurements to assess changes in the risk factors that have been commonly associated with low-back injury, that is, the cumulative low-back load, the peak low-back load and the flexion relaxation, or a subset of them (McGill and Kippers, 1994; Norman et al., 1998). Regarding upper-limb OEs, only 2 out of 37 lab and field studies extracted fatigue-related indexes, computed on EMG signals or through indirect physiological measurements, that have been associated with the risk of developing the impingement syndrome or rotator cuff injuries due to repetitive upper-limb tasks (Frost and Andersen, 1999; Grieve and Dickerson, 2008; Leong et al., 2019).

**Figure 3.**
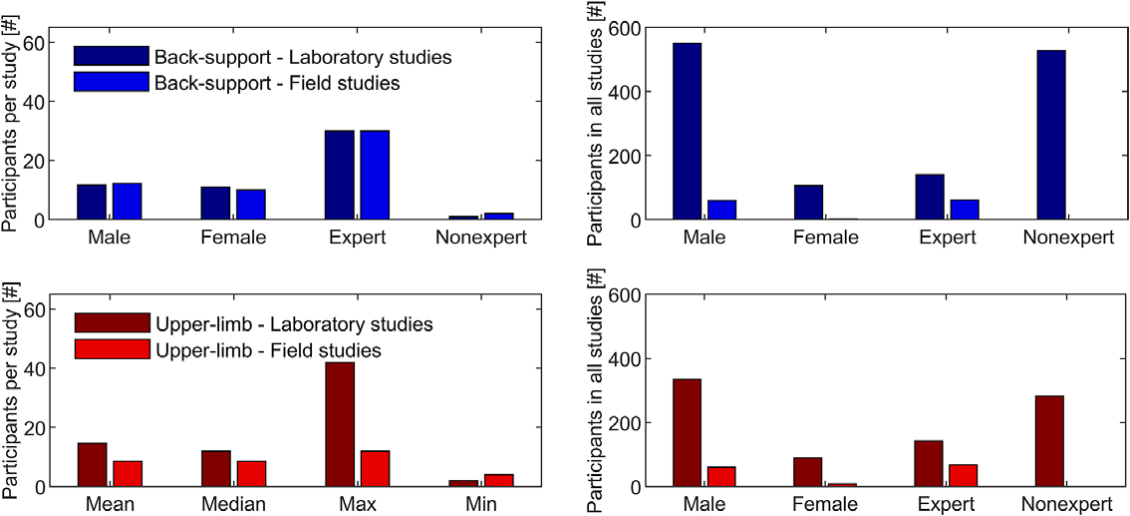
Sample size analysis of the studies using back-support and upper-limb exoskeletons.

The transfer of laboratory results to the field also depends on the subject group involved. Figure 3 presents the sample sizes from the selected papers. Novice participants or university students are mainly recruited in laboratory studies, whereas field studies include industrial workers, who have a routine in industrial tasks. In laboratory studies, the expert to nonexpert ratio is about 1:3.7 and 1:1.7 in the case of back-support and upper-limb exoskeletons, respectively. Furthermore, the average sample size is higher in laboratory studies than in field studies in the case of upper-limb exoskeletons a ratio of 1.7:1, whereas in the case of back-support exoskeletons the ratio is more balanced (1:1). Exoskeleton evaluations mainly target male participants whereas female end-users have been considered much less frequently.

Besides the physical impact of exoskeletons on the human body, their social impact is of major importance for the successful implementation at the workplace. A recent paper by Elprama et al. (2020) investigated the workers’ intention to use OEs (*n* = 124). A major gap in literature still exists regarding social measures, such as attitude toward exoskeletons and social perception of exoskeletons. Companies will only implement OEs on a mass scale if they effectively reduce physical and mental loads, and are socially accepted at individual and group levels. Furthermore, acute effects are abundantly researched, but no information is available on the long-term impact of exoskeleton use. Postmarket follow-up studies also allow for an investigation into the cost–benefit and cost-effectiveness of the initial investment.

**Figure 4.**
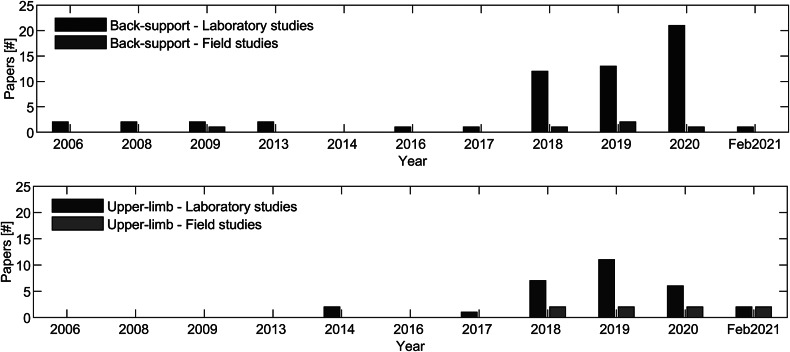
Number of papers that were published in the field of exoskeleton evaluations in laboratory and field conditions.

As visualized in Figure 4, a growing number of papers have been published in recent years that evaluate exoskeletons in laboratory conditions. Over 80% of the reviewed papers describe laboratory studies of back-support (*n* = 57) and upper-limb exoskeletons (*n* = 29). Minimum viable products and minimum marketable products are progressively being tested in field situations. Although around 15 exoskeletons are commercially available, only 12 of the identified papers provided insights into field evaluations of back-support (*n* = 5) and upper-limb exoskeletons (*n* = 8).

## Roadmap Toward Large-Scale Adoption of OEs

### Involving All Stakeholders

Despite the uptake in different domains of application, OEs are still in an early adoption stage. Although certain domains are ahead of others, with the manufacturing industry currently demonstrating the highest uptake, OEs still have not undergone a significant deployment. In this process, a major factor is that several stakeholders may have a different understanding of the costs (e.g., purchase costs and costs related to the loss of productivity due to training sessions) and benefits (e.g., increased occupational health of the workforce and increased productivity due to reduced fatigue and injuries) associated with the adoption of OEs, and may therefore have different opinions on whether and how the OEs can support their specific interests and goals.

As different stakeholders have different interests, they may need different types of information to build their opinion on OEs. Table 2 lists important stakeholders on the demand-side of OEs and indicates their specific goals and interests, that supposedly can be served by this technology, and what type of information they may use to develop their understanding and perspective. Obviously, also stakeholders on the supply side of OEs (such as manufacturers, technology developers, and researchers) will play an important role in creating this market, but, at this stage, it is expected that further advancements of the technology will mainly serve the fulfillment of the needs emerged from the end-users side. It is worth noticing that the information reported in Table 2 has been agreed upon by consensus between the authors and could be further refined by discussing with the stakeholders, with the objective to better define and prioritize the contents.

**Table 2.** List of stakeholders on the demand side involved in the process of adoption of occupational exoskeletons (OEs)

OE stakeholders	Key interests and goals	Type of information
Workers	Individual reduction of work-related fatigue and injury Workplace convenience: ease of use/usability (e.g., easy don-doff and maintaining the flexibility of movement) Actual and perceived performance Actual and perceived reduction of fatigue in their workstation	Summarized conclusions on impact on occupational health on short- and long-term Experience stories from diverse application domains First hand user experience (i.e., try-outs in own situation)
Unions and workers’ associations	Statistically significant reduction of work-related fatigue and injury Usability/acceptability in given workstations Potential and actual increase of productivity Actual and perceived reduction of fatigue in their workstation Capacity to improve ergonomics risk Clear boundary conditions required to make use of OEs successful Regulation of practices (e.g., voluntary/mandatory use and privacy issues related to the monitoring of behaviors) Regulation to assure safe and effective use of OEs	Transferrable knowledge on short−/long-term positive and adverse effects of using OEs on human biomechanics and health status obtained from short-term and long-term scientific studies Experience stories from diverse application domains Regulation specific for OEs Standards/guidelines assuring safety and performance of OEs Quality marks to distinguish good from bad products Ergonomic risk-assessment methods
Policy makers	Reduced healthcare expenses, increased industrial productivity Regulation to assure safe and effective use of OEs	Transferrable knowledge on short−/long-term positive and adverse effects of using OEs on human biomechanics and health status obtained from short-term and long-term scientific studies Standards/guidelines assuring safety and performance of OEs Economic analyses of cost and benefit of applying OEs
Ergonomists, kinesiologists, occupational medical doctors, and HSE	Comprehensive knowledge to advise on and select OEs for specific applications or work situations Applicable methodologies for computation of ergonomic risk indexes to account for the use of OEs Any relevant psychological implications connected to the use of OEs Working principles of OEs, how OEs are operated, maintained and sanitized Regulation of practices (e.g., voluntary/mandatory use and privacy issues related to the monitoring of behaviors)	Transferrable knowledge on short−/long-term effect of using OEs on human biomechanics and health status obtained from short-term and long-term scientific studies Experience stories from diverse application domains Regulations specific to OEs Standards/guidelines assuring safety and performance of OEs Quality marks to distinguish good from bad products Ergonomic risk-assessment methods
Corporate management, company’s decision-makers	Potential and actual increase of capacity and productivity, due to reduced fatigue and injuries Governmental support (e.g., fiscal credit) for the use of OEs Regulation of practices (e.g., voluntary/mandatory use and privacy issues related to monitoring of behaviors) Cost/benefit ratio, considering all factors, including changes to organizational models and logistics of the workplace and processes	Summarized conclusions on impact on occupational health on short- and long-term Summarized conclusions on cost/benefit of using OEs Information on impact on company’s organizational models and logistics to comply with the adoption of OEs (e.g., dedicated rooms to store/sanitize OEs) Experience stories from diverse application domains Regulations specific to OEs Standards/guidelines supporting the adoption of OEs Quality marks to distinguish good from bad products
Insurance companies	Regulation to assure safe and effective use of OEs Reducing healthcare expenses related to occupational health	Comprehensive knowledge on short-/long-term effect of using OEs on human biomechanics and health status obtained from short-term and long-term scientific studies Standards/guidelines supporting the adoption of OEs Quality marks to distinguish good from bad products Ergonomic risk-assessment methods Economic analyses of cost and benefit of applying OEs
Certifying bodies	Publishing standardized approaches to demonstrating performance and safety of OEs Publishing standards on use practice of OEs (user guidelines)	Scientific knowledge on factors determining performance and safety of OEs Detailed information on tests performed in practice and specific outcomes and feasibility information

*Note:* For each stakeholder, the key interests, goals, and information needs are listed. The information reported in this table has been agreed upon by consensus between the authors.

The list of stakeholders reported in Table 2 highlights the need to build a comprehensive scientific body of knowledge on the effects of OEs on occupational health (from their impact on fatigue and injury to occupational health). This need requires field studies and a variety of measurements spanning from biomechanics and performance, to usability, acceptability, and user experience. Such a holistic assessment would provide evidence of the benefits as well as possible undesired effects of OEs on human health, which would be the first fundamental step to designing novel regulated practices, standards, or methods for ergonomic risk assessment. It is important to note that prevention of health issues typically is a combination of private and public interests, which is reflected in the existing practice around ergonomic and safety-related tools and procedures. Often, guidelines that prescribe what is needed or required in specific sites are based on nonconclusive evidence, through experience-based guidelines, such as the NIOSH lifting equation, or the OCRA-checklist. Such existing frameworks do not yet incorporate a reflection on how OEs interact with such guidelines. In this regard, the release of a new guideline of the Ergonomic Assessment Work-Sheet (EAWS), the EXO-EAWS,^2^ appears to represent an important milestone in the field since this method considers the possibility that the risk assessment index changes with the use of a certified occupational exoskeleton for the first time. Similarly, Di Natali et al. (2021) proposed a new method to quantify the ergonomic benefit introduced by using a low-back exoskeleton in lifting tasks.

**Figure 5.**
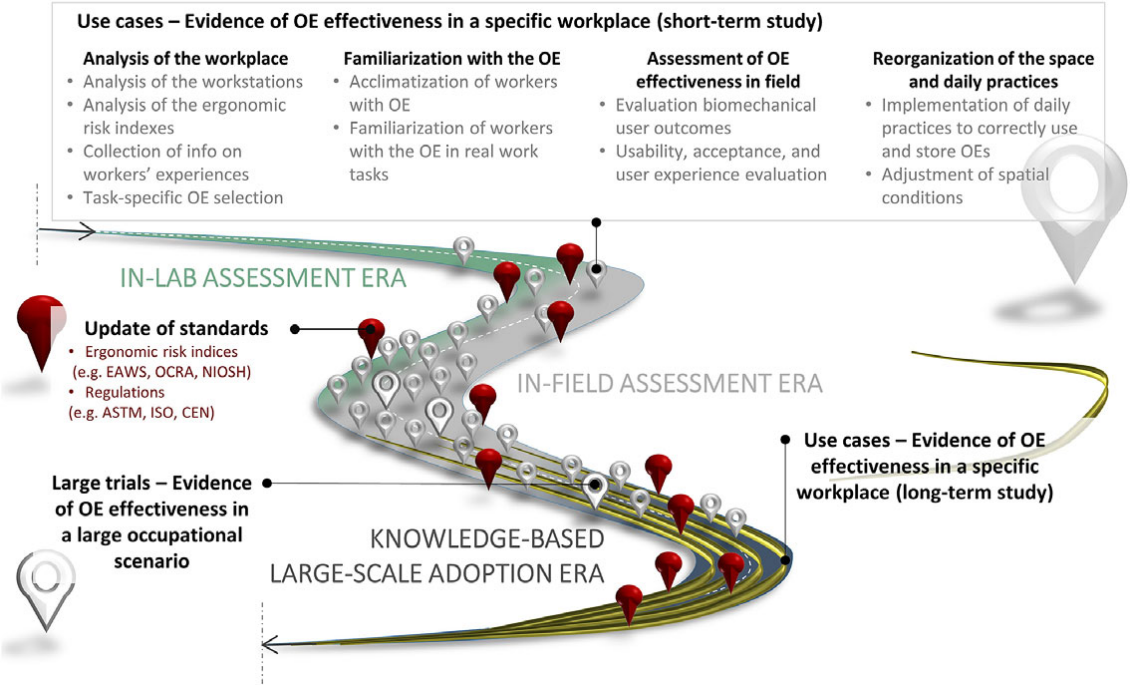
Roadmap toward large-scale adoption of OEs.

Based on the above considerations, Figure 5 represents a proposed roadmap envisioned by the authors toward the large-scale adoption of OEs. While in the past years, most of the scientific literature has focused on laboratory studies to verify the main biomechanics effects of OEs on the human body (as depicted in S ection “Scientific Evidence of OE Effectiveness in Laboratory and Field Studies”), we believe that in the future, scientific research should more consistently and considerably focus on bringing OEs out of laboratories and testing them in field scenarios. By capturing evidence of effectiveness in several short-term field studies and by using biomechanical measures for the assessment of risk factors for injury, it will become possible to collect success stories from various use cases, which could be the starting point for designing larger-scale studies that can help generalize the findings collected in a specific workplace to a broader application scenario. While, without exoskeletons, large field studies have been carried out to assess the risk of injury at workplaces (Norman et al., 1998), the state-of-the-art of methodological aspects of evaluating OEs reported in Section “Scientific Evidence of OE Effectiveness in Laboratory and Field Studies” highlights the lack of large-scale studies, as sample sizes are in most cases limited and the generalizability of the results is difficult in almost all application scenarios.

Notably, various multinational companies, especially in the automotive field, are currently running large OEs evaluation tests within their plants. However, despite very limited communications at conferences and events, the testing methodology and results of those studies with OEs do not appear to have been presented in scientific publications yet. While it may be possible that companies have limited interest in sharing their data with the scientific community or competitors for strategic reasons, in our opinion the cooperation between companies and the academic/scientific community is paramount for building a solid body of scientific literature on OEs that can lead to the large-scale adoption of this technology in various application scenarios. In addition, workplace insurance boards may play an important role to pursue this type of study, having both the funding and incentive to assess whether the technology can impact the risk of injury, similar to large-scale studies carried out (without OEs) to assess the risk of injury at the workplace (Norman et al., 1998).

Finally, the output of large field studies may support the development of clear indications about the regulatory context for OEs. Currently, OEs could be classified as medical or nonmedical devices, but a consensus on which choice is more suitable has not been reached yet by the community. In principle, this depends on the intended application domain and claims used on performance and impact of the product, and therefore remains a choice of the manufacturer; nevertheless, this choice is also directed by the expectations of other stakeholders. Some commercial passive OEs have been certified as class I medical devices, according to the European Directive 93/42/EEC, whereas others have been certified as work equipment according to the European directive 2009/104/EC. To the authors’ knowledge, no commercial devices have been certified according to the European Regulation 2016/425 (i.e., the directive on personal protective equipment) to date, but the use of OEs as personal protective equipment has been proposed on several occasions and some attempts have been made to investigate the matter in the United States (Butler and Gillette, 2019). The different approaches followed by OE manufactures reflect the lack of clear indications of which specific requirements an OE should meet for safe and effective implementation.

### Building Evidence Through Considering Use Cases

Although relatively few field studies of OEs have been performed to date, a progressive increase in their number has been noted (Figure 5). However, the variety of methodological approaches used for OE testing (in terms of workstations, experimental methods, metrics, and assessment of risk factors for injury) makes the findings difficult to compare and summarize. In the authors’ opinion, the decision of adopting OEs within a plant requires a stepwise knowledge-based approach, from the observation of the workers’ tasks and routines (to identify the technology that most suits their needs), to the familiarization with and use of the technology (for the assessment of the users’ experience), and up to the final reorganization of the plant space and daily routine to support the adoption of the technology (Figure 5; Masood et al., 2019; Kerangueven and Atain-Kouadio, 2020). First of all, understanding the *needs* of the workers in each specific workplace is a complex yet necessary process for a successful deployment of technology. Aiming at user acceptance in daily practice, identifying the workers’ needs requires use-case-specific *effectiveness* metrics (Sylla et al., 2014; Otten et al., 2018; Torricelli et al., 2020). In terms of user-centered design approaches, these could be used to develop customized technology and to evaluate competing commercially available solutions to find the most promising for each use case. Observing the workers’ tasks in their daily practice is paramount to collecting information on which repetitive and nonrepetitive actions are performed (Sylla et al., 2014). This facilitates collecting the users’ opinions on their perceived physical effort required to perform strenuous or repetitive actions, which might later be complemented by reports about their experience of the exoskeleton (Shore et al., 2020).

Furthermore, analyzing the interaction of a worker with the environment is of high importance: several workers might be confined to work in narrow spaces (such as in-car interior assembly), very close to colleagues (such as during handling, cutting, or storing food pieces), or in complex or unstructured environments (such as outdoor gardening or painting). Context-specific information can be complemented by ergonomics risk assessment with well-recognized methods such as OCRA (Occhipinti, 1998), EAWS (Schaub et al., 2012), or NIOSH (Dempsey, 2002). This helps to quantitatively evaluate the effect of repetitive or strenuous actions, and to assess the risk level of the workstation or task under consideration, which could complement the historical data of the companies on the correlation between the development of work-related MSDs and specific workstations.

Analyzing the users’ routines and interaction with the environment, and quantifying ergonomics risks constitutes the basis for evaluating apriori if an OE would have a significant positive impact on the worker’s job quality and health. Such an apriori assessment of the workstation is crucial, as exoskeletons use should only be recommended when indications of the real advantages are clear before any experimentation is performed. This would spare the efforts of testing OEs for situations that do not allow successful exoskeleton implementation, for example, when only a small portion of the time is spent performing the supported movement, or when the movements are highly variable and complex, or when the environment does not allow for a safe introduction of additional tools.

After the deployment, the assessment of OE *effectiveness* requires, again, a holistic approach (Howard et al., 2020). While a lot of attention has been put on assessing biomechanical effects, particularly for the target movement, effectiveness seems to be distinctly influenced by psychological and usability factors. Analyzing these calls for further metrics to assess the worker’s experience (Beckerle et al., 2017; Torricelli et al., 2020), using psychometric techniques, for example, questionnaires (Hart and Staveland, 1988; Minge et al., 2017) or psychophysiological methods, for example, pupil size, heart rate, and skin conductivity (Armel and Ramachandran, 2003; Schultheis and Jameson, 2004; Ikehara and Crosby, 2005). Separate to biomechanical metrics, usability and acceptance can capture aspects that relate to the environment and the organization of the work related to the use of an OE (such as indications for daily practice of effective wearing, storing, and sanitization activities).

Given the above considerations on pre- and post-deployment analyses, the results of the *effectiveness* evaluation of an OE appear very use-case-specific. Hence, a debate within the scientific community could make it possible to generalize the results of an OE evaluation for specific use-cases to similar job tasks in diverse conditions. Considering that exoskeleton technology is not yet fully mature, many early adopters are cautiously evaluating the OE effectiveness at their specific tasks, which hampers answering this open question. Testing OEs with rigorous methods will certainly help collect information on suitable jobs where the apriori analysis, users’ feedback, and cost-effectiveness agree on their value.

If the testing phase leads to positive results, the decision of the company to adopt this technology requires relatively simple yet necessary modifications of spaces and daily practices to ensure that workers are properly instructed on how to use, store and sanitize the device (similar to personal protective equipment). The reorganization of spaces involves installing dedicated changing rooms or exoskeletons hangers to store the devices. From the perspective of daily routines, the organization of periodic training courses is necessary to instruct and monitor the workers on the correct use, storage, sanitation, and maintenance of the devices. Also, clear indications of when and how to use the exoskeletons during the shift should be provided for the workers and considered in the method-time-measurement analysis of the production line. The decision to introduce exoskeletons into a production line necessarily involves several departments of the company, for example, the health and safety, human resources, and production departments, among others. In medium and large companies, the involvement of unions in the decision process would also ensure that the workers understand this change as an improvement of their health conditions at work.

Finally, in the authors’ opinion, large-scale and longer-term field studies should complement detailed laboratory studies to monitor the effects of daily use of exoskeletons, and particularly to verify the causal relationship between the reduced physical loading of the body and the occurrence of specific work-related MSDs. Such studies can help identify, monitor, and quantify the possible undesired side-effects related to exoskeleton use (Howard et al., 2020), as well as provide solid scientific knowledge to support the revision of ergonomics risk-assessment methods, the definition of clear guidelines for selecting OEs (such as quality marks to distinguish between good or bad technologies), practices to use them, and the revision of the related safety standards and regulations.

## Conclusion

Considering that OEs are still in an early adoption stage, large-scale deployment of this technology will necessarily go through a more structured involvement of all stakeholders, such as the workers, the health and safety, human resources and production departments, as well as the unions and the policymakers. All stakeholders should be informed of the costs and benefits of OEs from their perspectives and interests, as they can have different objectives and therefore require different knowledge to make their decisions. From this context, it is important to consider larger field studies not just as scientific studies, but also as experiments-in-practice, that allow all stakeholders to determine and develop their understanding. It is therefore of key importance that all relevant stakeholders are to some degree involved in the definition of any larger studies in the field, in order to maximize the impact of the studies on an eventual larger uptake of OEs.

The large-scale adoption of OEs will require a stepwise knowledge-based approach, grounded on careful investigation of work task biomechanics, usability, acceptability, and user experience. Thus, we believe that in the future, scientific research will need to consistently focus on complementing laboratory studies with field investigations. By capturing evidence of effectiveness in several use cases, through short-term field studies, it will become possible to start designing larger-scale studies. Such studies are of paramount importance since they could help to generalize the findings collected in specific workplaces to broader application scenarios. Finally, longer-term field studies should be carried out with the key objective to verify whether OEs can reduce the occurrence of specific work-related MSDs.

## Data Availability

Data sharing is not applicable to this article as no new data were created or analyzed in this study.

## Footnotes

1
www.cdc.gov/workplacehealthpromotion/health-strategies/musculoskeletal-disorders/index.html

2
www.eaws.it

## Supplementary material

For supplementary material accompanying this paper visit https://doi.org/10.1017/wtc.2021.11. click here to view supplementary material

## Abbreviations

EMG: electromyography
OE: occupational exoskeleton
RPE: rate of perceived exertion
SUS: system usability scale
WRMSDs: work-related musculoskeletal disorders
