# Supplementary material for: Occupational exoskeletons: A roadmap toward large-scale adoption. Methodology and challenges of bringing exoskeletons to workplaces
Source: Wearable Technol. 2021 Sep 17;2:e11. doi: 10.1017/wtc.2021.11 (PMC10936259; doi:10.1017/wtc.2021.11)
Supplement: Supplementary file 1 [file S2631717621000116sup001.docx]

<short running-title>

<author-name details>

Occupational Exoskeletons: A Roadmap Towards Large-Scale Adoption

Methodology and challenges of bringing exoskeletons to workplaces

Simona Crea^1,2,§^, Philipp Beckerle^3,4,^*, Michiel De Looze^5,^*, Kevin De Pauw^6,^*, Lorenzo Grazi^1,^*, Tjaša Kermavnar^7,^*, Jawad Masood^8,^*, Leonard W. O’Sullivan^7,^*, Ilaria Pacifico^1,^*, Carlos Rodriguez-Guerrero^9,^*, Nicola Vitiello^1,2,^*, Danijela Ristić-Durrant^10,§^, Jan Veneman^11,§^

^1^Scuola Superiore Sant’Anna, The BioRobotics Institute, viale Rinaldo Piaggio 34, 56025, Pontedera, Italy

^2^IRCCS Fondazione Don Gnocchi, Via di Scandicci 269, 50143, Florence, Italy

^3^Chair of Autonomous Systems and Mechatronics, Friedrich-Alexander-Universität Erlangen-Nürnberg, Paul-Gordan-Straße 3/5, 91052, Erlangen, Germany

^4^Institute for Mechatronic Systems, Technische Universität Darmstadt, Otto-Berndt-Straße 2, 65287, Darmstadt, Germany

^5^TNO, Schipholweg 77, 2316 ZL, Leiden, The Netherlands

^6^Human Physiology and Sports Physiotherapy research group, and Brussels Human Robotics Research Center (BruBotics), Vrije Universiteit Brussel, Pleinlaan 2, 1050 Brussels, Belgium

^7^School of Design, and Confirm Smart Manufacturing Centre, University of Limerick, Castletroy, Limerick, V94 T9PX, Co. Limerick, Ireland

^8^Processes and Factory of the Future Department, CTAG – Centro Tecnológico de Automoción de Galicia, Polígono Industrial A Granxa, O Porriño, 36475, Pontevedra, Spain

^9^Robotics and Multibody Mechanics Research Group, Department of Mechanical Engineering, Vrije Universiteit Brussel and Flanders Make, Pleinlaan 2, Brussel 1050, Belgium

^10^Institute of Automation, University of Bremen, Otto-Hahn-Allee 1, 28359 Bremen, Germany

^11^Hocoma AG, Industriestrasse 4, 8604, Volketswil, Zürich, Switzerland

*Alphabetic order

^§^Corresponding authors: [simona.crea@santannapisa.it](mailto:simona.crea@santannapisa.it); [ristic@iat.uni-bremen.de](mailto:ristic@iat.uni-bremen.de); [jan.veneman.cost@gmail.com](mailto:jan.veneman.cost@gmail.com)

Received:----; Revised:----; Accepted:-----;

# Supplementary materials

Table 3. List of in-lab studies on upper-limb exoskeletons.

| **Study** | **Exoskeletons** | **Exoskeletons’ exploitation level** | **Subjects’ number and gender** | **Subjects’ level of experience** | **Biomechanical risk-related indexes** |
| --- | --- | --- | --- | --- | --- |
| *Alabdulkarim et al., 2019* | - Fortis, - ShoulderX, - Fawcett Exovest with zero-gravity arm | Market | 16 (8 male, 8 female) | Non-expert | n/a |
| *Blanco et al., 2019* | MovilFrio | Prototype | 10 (8 male, 2 female) | Non-expert | n/a |
| *Blanco et al., 2020* | ExIF project | Prototype | 12 (11 male, 1 female) | Non-expert | n/a |
| *de Vries et al., 2019* | Skelex | Market | 12 male | Non-expert | Total moment at the shoulder joint |
| *de Vries et al., 2021* | Skelex | Market | 11 male | Expert | n/a |
| *Grazi et al., 2020* | H-PULSE | Prototype | 10 male | Non-expert | n/a |
| *Huysamen et al., 2018a* | Robo-Mate | Prototype | 8 (4 male, 4 female) | Non-expert | n/a |
| *Hyun et al., 2019* | H-VEX | Pre-Market | 10 male | Non-expert | n/a |
| *Kelson et al., 2019* | - EksoVest, - Levitate Airframe | Market | 11 (7 male, 4 female) | Non-expert | n/a |
| *Kim et al., 2019* | EksoVest | Market | 12 (6 male, 6 female) | Non-expert | n/a |
| *Kim et al., 2018a* | Proto-EksoVest | Pre-Market | 12 (6 male, 6 female) | Non-expert | n/a |
| *Kim et al., 2018b* | Proto-EksoVest | Pre-Market | 27 (14 male, 13 female) | Non-expert | n/a |
| *Maurice et al., 2019* | Paexo | Market | 12 male | Non-expert | n/a |
| *Moyon et al., 2019* | Skelex | Market | 36 (18 male, 18 female) | Non-expert | n/a |
| *Otten et al., 2018* | Lucy 2.0 | Prototype | 3 (2 male, 1 female) | Non-expert | n/a |
| *Pacifico et al., 2020* | Proto-MATE | Pre-Market | 15 male | Non-expert | n/a |
| *Perez Luque et al., 2020* | - EksoVest, - Paexo, - MATE | Market | 17 (11 male, 6 female) | 8 expert, 9 non-expert | n/a |
| *Pinho et al., 2020* | - ShoulderX, - MATE, - Paexo | Market | 2 male | Expert | n/a |
| *Rashedi et al., 2014* | WADE | Prototype | 12 male | Non-expert | n/a |
| *Schmalz et al., 2019a* | Paexo | Market | 12 (6 male, 6 female) | Non-expert | n/a |
| *Schmalz et al., 2019b* | Paexo | Market | 12 (6 male, 6 female) | Non-expert | n/a |
| *Spada et al., 2018a* | Levitate Airframe | Market | 42 male | Expert | n/a |
| *Spada et al., 2017* | Levitate Airframe | Market | 29 male | Expert | n/a |
| *Spada et al., 2018b* | Proto-MATE | Pre-Market | 18 male | Expert | n/a |
| *Sylla et al., 2014* | ABLE | Prototype | 8 male | Non-expert | Sum of joint torques |
| *Theurel et al., 2018* | EXHAUSS Stronger | Prototype | 8 (4 male, 4 female) | Non-expert | n/a |
| *Van Engelhoven et al., 2019* | ShoulderX | Market | 14 male | Expert | n/a. |
| *Wang et al., 2021* | PULE | Prototype | 18 male | Expert | n/a |
| *Yin et al., 2020* | PULE | Market | 15 male | Non-expert | n/a |

Table 4. List of in-field studies on upper-limb exoskeletons.

| **Study** | **Exoskeletons** | **Exoskeletons’ exploitation level** | **Subjects’ number and gender** | **Subjects’ level of experience** | **Biomechanical risk-related indexes** |
| --- | --- | --- | --- | --- | --- |
| *De Bock et al., 2020* | - ShoulderX, - Skelex | Market | 4 male | Expert | n/a |
| *Gillette & Stephenson, 2019* | Levitate Airframe | Market | 6 (4 male, 2 female) | Expert | n/a |
| *Gillette & Stephenson, 2018* | Levitate Airframe | Market | 11 male | Expert | n/a |
| *Hefferle, et al., 2021* | Crimson Dynamics,  Skelex V1 | Market | 8 male | Expert | n/a |
| *Iranzo et al., 2020* | Levitate Airframe | Market | 12 (11 male, 1 female) | Expert | n/a |
| *Moyon et al., 2018* | Skelex | Market | 9 (5 male, 4 female) | Expert | n/a |
| *Smets et al., 2019* | EksoVest | Market | 10 (9 male, 1 female) | Expert | n/a |
| *Wang et al., 2021* | PULE | Prototype | 8 male | Expert | n/a |

Table 5. List of in-lab studies on back-support exoskeletons.

| **Study** | **Exoskeletons** | **Exoskeletons’ exploitation level** | **Subjects’ number and gender** | **Subjects’ level of experience** | **Biomechanical risk-related indexes** |
| --- | --- | --- | --- | --- | --- |
| *Abdoli-E et al., 2006a* | - PLAD | Prototype | 9 male | Non-expert | n/a |
| *Abdoli-E et al., 2008* | - PLAD | Prototype | 9 male | Non-expert | Integrated moments about L4/L5 joint |
| *Abdoli-E et al., 2006b* | - PLAD | Prototype | 9 male | Non-expert | Percentage of compression and shear reduction on L4/L5 joint  Peak reaction force and pressure on L4/L5 joint |
| *Alemi et al., 2019* | - VT-Lowe | Prototype | 12 male | Non-expert | n/a |
| *Alemi et al., 2020* | - BackX, - Laevo | Market | 18 (9 male, 9 female) | Non-expert | n/a |
| *Baltrusch et al., 2020a* | - SPEXOR | Prototype | 17 male | Expert | Knee, hip, L5/S1 joints mechanical work |
| *Baltrusch et al., 2020b* | - SPEXOR | Prototype | 10 male | Expert | n/a |
| *Baltrusch et al., 2020c* | - SPEXOR | Prototype | 24 male | Expert | n/a |
| *Baltrusch et al., 2018a* | - Laevo | Market | 18 male | Non-expert | n/a |
| *Baltrusch et al., 2018b* | - Laevo | Market | 18 male | Non-expert | n/a |
| *Baltrusch et al., 2019* | - Laevo | Market | 18 male | Non-expert | n/a |
| *Bosch et al., 2016* | - Laevo | Market | 18 male (9 male, 9 female) | Non-expert | n/a |
| *Chen et al., 2018* | - APO | Prototype | 7 male | Non-expert | n/a |
| *Dewi et al., 2018* | - PAS | Prototype | 13 (7 male, 6 female) | Expert | n/a |
| *Frost et al., 2009* | - PLAD | Prototype | 13 male | Non-expert | Integrated L4/L5 joint moment |
| *Goršič et al., 2019* | - Modular reconfigurable trunk exoskeleton | Prototype | 12 (11 male, 1 female) | Non-expert | n/a |
| *Han et al., 2019* | - Exoskeleton with passive storage mechanism | Prototype | 3 | Non-expert | n/a |
| *Heo et al., 2020* | - Pneumatic back-support exoskeleton | Prototype | 10 male | Non-expert | n/a |
| *Hussain et al., 2020* | - Exoskeleton for lifting | Prototype | 10 (7 male, 3 female) | Non-expert | n/a |
| *Huysamen et al., 2018b* | - Robo-Mate | Prototype | 12 male | Non-expert | Contact pressure |
| *Hyun et al., 2020* | - H-WEX V2 | Prototype | 10 male | Non-expert | n/a |
| *Inose et al., 2017* | - AB-Wear | Prototype | 1 male | Non-expert | n/a |
| *Kazerooni et al., 2019* | - BackX | Market | 8 (4 male, 4 female) | Non-expert | n/a |
| *Kim et al., 2020* | - BackX, - Laevo V2.5 | Market | 18 (9 male, 9 female) | Non-expert | n/a |
| *Ko et al., 2018* | - H-WEX | Prototype | 9 male | Non-expert | n/a |
| *Kobayashi et al., 2008* | - Muscle Suit | Prototype | 3 male | Non-expert | n/a |
| *Koopman et al. 2020a* | - SPEXOR | Prototype | 10 male | Expert | Compression force and moment at L5/S1 joint |
| *Koopman et al., 2019a* | - Laevo | Market | 11 male | Non-expert | Net L5/S1 moment |
| *Koopman et al., 2020b* | - Laevo V2.4 | Market | 11 male | Non-expert | Peak compression forces on L5/S1 disc  Peak L5/S1 joint moment |
| *Koopman et al., 2019b* | - Mk2B (Robo-Mate 2^nd^ version) | Prototype | 10 male | Non-expert | Peak compression forces on L5/S1 disc  Peak L5/S1 joint moment |
| *Kozinc et al., 2020a* | - SPEXOR | Prototype | 20 (10 male, 10 female) | Non-expert | n/a |
| *Kozinc et al., 2020b* | - SPEXOR | Prototype | 14 (7 male, 7 female) | Non-expert | n/a |
| *Lamers et al., 2018* | - Garment with elastic bands | Prototype | 8 (7 male, 1 female) | Non-expert | Intervertebral compression disc (estimate) |
| *Lanotte et al., 2018* | - APO | Prototype | 5 male | Non-expert | n/a |
| *Lazzaroni et al., 2019* | - Robo-Mate | Prototype | 7 male | Non-expert | n/a |
| *Lotz et al., 2009* | - PLAD | Prototype | 10 male | Non-expert | n/a |
| *Luo et al., 2013* | - WSAD | Prototype | 1 male | Non-expert | n/a |
| *Madinei et al., 2020a* | - BackX, - Laevo | Market | 18 (9 male, 9 female) | Non-expert | n/a |
| *Madinei et al., 2020b* | - BackX, - Laevo | Market | 18 (9 male, 9 female) | Non-expert | n/a |
| *Miura et al., 2020a* | - HAL for Care Support | Market | 19 (16 male, 3 female) | Non-expert | n/a |
| *Miura et al., 2020b* | - HAL for Care Support | Market | 18 (11 male, 7 female) | Non-expert | n/a |
| *Naf et al, 2018* | - SPEXOR | Prototype | 3 male | Non-expert | n/a |
| *Omoniyi et al., 2020* | - Laevo | Market | 15 (14 male, 1 female) | Expert | n/a |
| *Picchiotti et al., 2019* | - StrongArm Technologies FLx, - StrongArm Technologies V22 | Market | 10 male | Non-expert | Peak sagittal moment on L5/S1 joint  Spinal loading for compression and shear at L3/L4 and L5/S1 joints |
| *Poliero et al., 2020* | - XoTrunk | Prototype | 9 male | Non-expert | n/a |
| *Qu et al., 2021* | - IPAE | Pre-market | 8 male | Expert | n/a |
| *Shin et al., 2019* | - Pneumatic muscle-based back assistance exoskeleton | Prototype | 1 male | Non-expert | n/a |
| *So et al., 2020* | - BackX | Market | 30 (20 male, 10 female) | Expert | n/a |
| *Tan et al., 2019* | - HAL for Care Support | Market | 20 (13 male, 7 female) | Non-expert | n/a |
| *Thamsuwan et al., 2020* | - Laevo | Market | 14 (13 male, 1 female) | Expert | n/a |
| *Toxiri et al., 2018* | - Robo-Mate | Prototype | 11 male | Non-expert | n/a |
| *von Glinski et al., 2019* | - HAL for Care Support | Market | 14 male | Non-expert | n/a |
| *Wei et al., 2020* | - MeBot-EXO | Prototype | 7 male | Non-expert | n/a |
| *Weston et al., 2018* | - Steadicam Fawcett Exoskeletal vest | Market | 12 male | Non-expert | Compression and shear forces on L4/L5 and L5/S1 joints |
| *Whitfield et al., 2013* | - PLAD | Prototype | 15 male | Non-expert | n/a |
| *Yong et al., 2019* | - SIAT waist exoskeleton | Prototype | 10 | Non-expert | n/a |
| *Zhang et al., 2018* | - Lower back robotic exoskeleton | Prototype | 1 male | Non-expert | n/a |

Table 6. List of in-field studies on back-support exoskeletons.

| **Study** | **Exoskeletons** | **Exoskeletons’ exploitation level** | **Subjects’ number and gender** | **Subjects’ level of experience** | **Biomechanical risk-related indexes** |
| --- | --- | --- | --- | --- | --- |
| *Amandels et al., 2018* | - Laevo | Market | 9 male | Expert | n/a |
| *Graham et al., 2009* | PLAD | Prototype | 10 (8 male, 2 female) | Expert | Compression normalized EMG for spinal compression |
| *Hensel & Keil, 2019* | - Laevo | Market | 30 male | Expert | n/a |
| *Motmans et al., 2019* | - Laevo V2.5 | Market | 10 male | Expert | n/a |
| *Settembre et al., 2020* | Laevo | Market | 2 male | Expert | n/a |
